# Supplementary material for: Leucine Repeat Rich Kinase 1 Controls Osteoclast Activity by Managing Lysosomal Trafficking and Secretion
Source: Biology (Basel). 2023 Mar 29;12(4):511. doi: 10.3390/biology12040511 (PMC10135754; doi:10.3390/biology12040511)
Supplement: Supplementary file 1 [file biology-12-00511-s001.zip › biology-2280887-supplementary.pdf]

SM1

Original Blot Images for Figure 3B

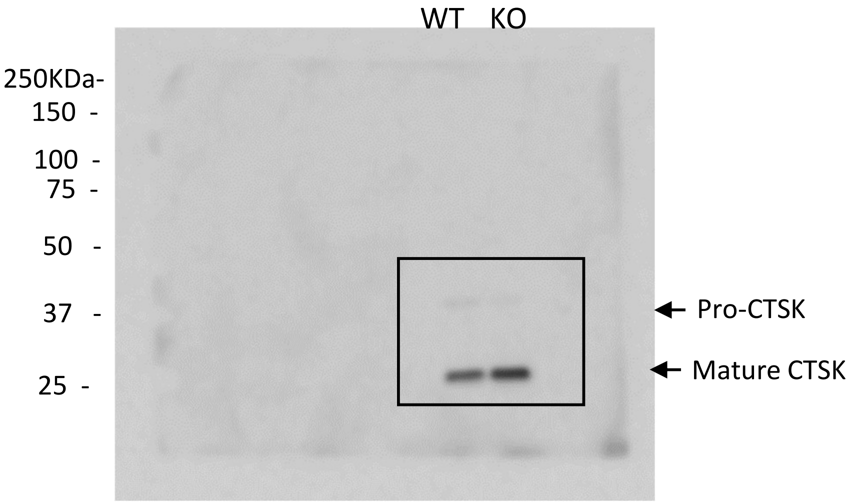

Blot to CTSK Ab
(the 46kDa pro-CTSK and the 27kDa mature CTSK were detected)

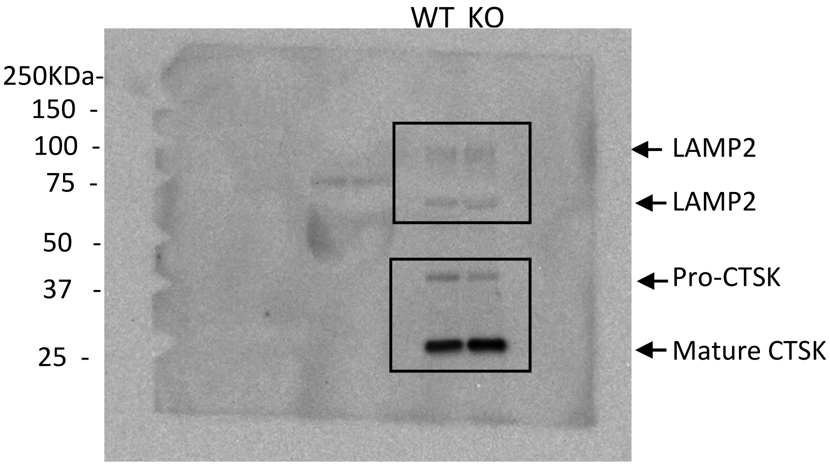

Blot to LAMP2 Ab after blotting to CTSK Ab
(multiple bands of the glycosylated LAMP2 around 70, 100-120kDa were detected)

SM2

Original Blot Images for Figure 3B (continue)

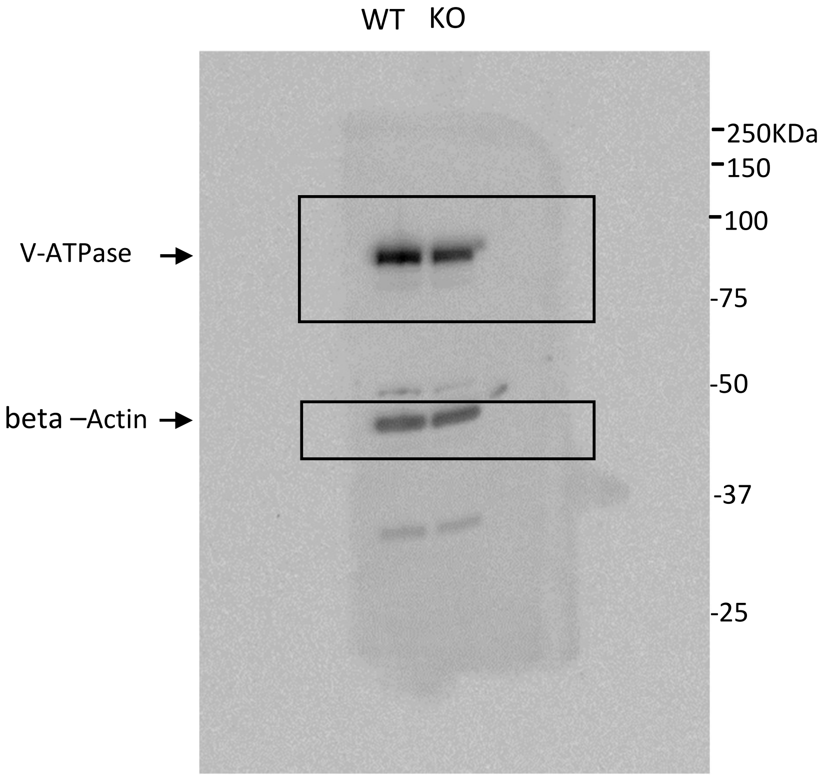

Blot to V-ATPase Ab (93 Kd) after blotting to beta-Actin Ab and CTSK Ab. Other Bands are CSTK.

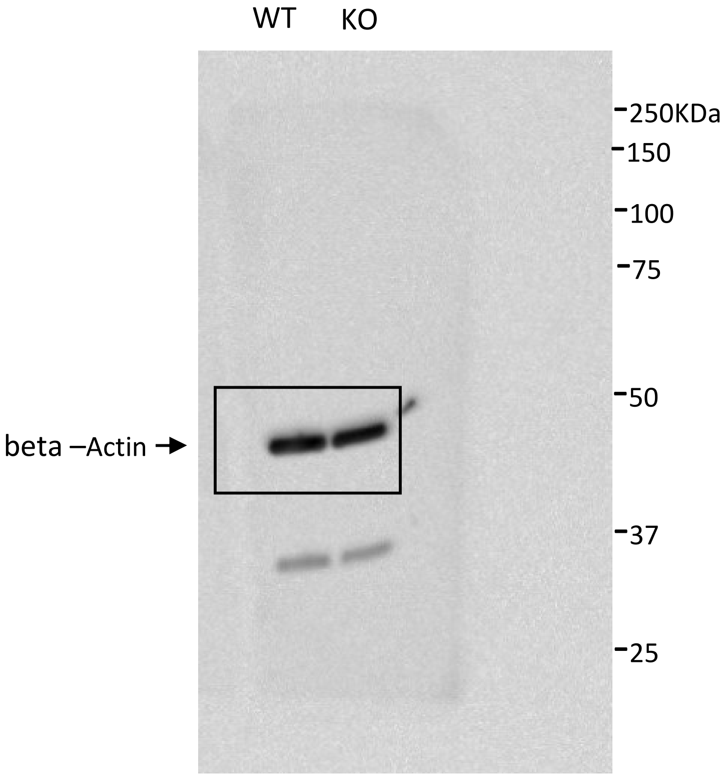

Blot to beta –Actin Ab after blotting to CTSK Ab. Other Bands are CSTK.

SM3

Original Blot Images for Figure 4B

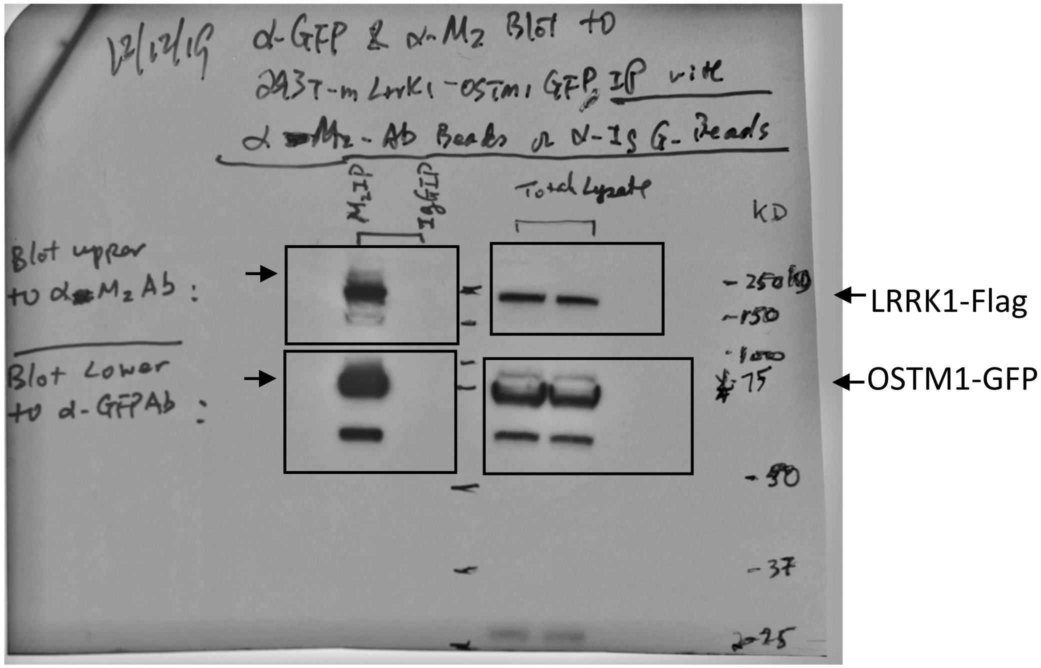

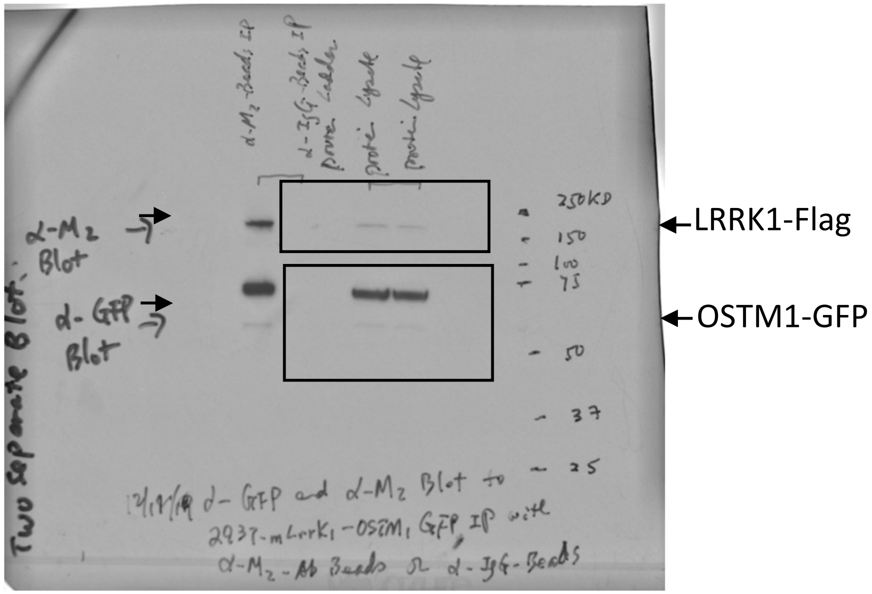

Blot to M2 Flag ab to detect LRRK1-Flag (upper panels)
Blot to GFP ab to detect OSTM1-GFP (lower panel)
Exposed to X-ray film for 1 minute

Blot to M2 Flag ab to detect LRRK1-Flag (upper panels)
Blot to GFP ab to detect OSTM1-GFP (lower panel)
Exposed to X-ray film for 10 seconds

SM4

Original Blot Images for Figure 4C

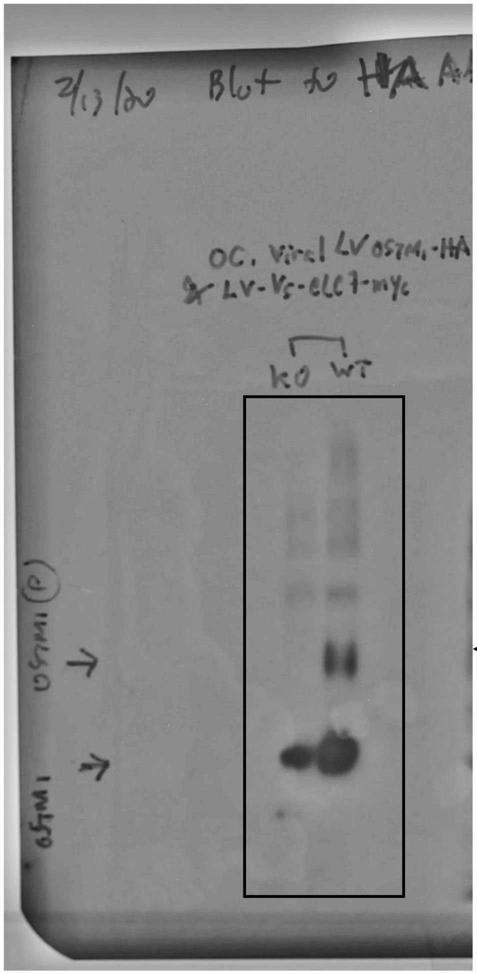

← Phosphorylated OSTM1

←OSTM1

←Degraded OSTM1

Blot to HA antibody to detect OSTM1-HA fusion protein
(No protein size marker was used on Phos-tag SDS-PAGE gel)

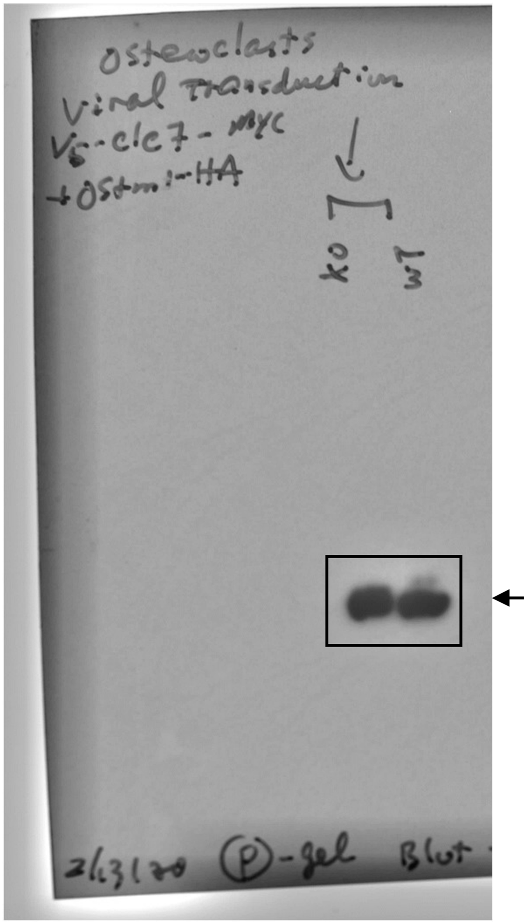

← beta-Actin

Blot to beta-Actin Ab
(No protein size marker was used on Phos-tag SDS-PAGE gel)

SM5

Original Blot Images for Figure 5A

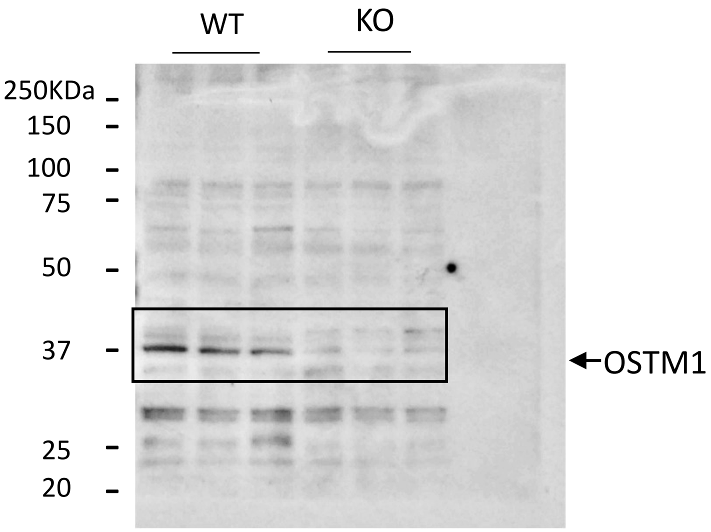

Blot to Ostm1 Ab

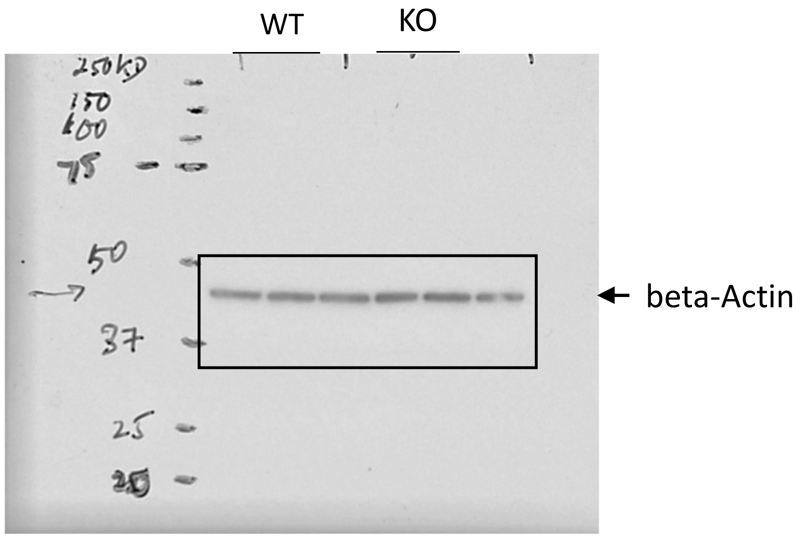

Blot to beta-Actin Ab
